# Supplementary material for: Predicting visual function by interpreting a neuronal wiring diagram
Source: Nature. 2024 Oct 2;634(8032):113–23. doi: 10.1038/s41586-024-07953-5 (PMC11446822; doi:10.1038/s41586-024-07953-5)
Supplement: Supplementary file 6 — CRF and ERF predictions for individual TmY4 and TmY9 cells. Analogous to Supplementary Data 3, but for TmY target types. Shown are the top four monosynaptic pathways, the strongest pathway passing through each of the top ten intermediary types (ranking from Extended Data Fig. 7), and the trisynaptic pathway Tm1–TmY–Dm3–TmY (see the section entitled Prediction of spatial normalization). [file 41586_2024_7953_MOESM6_ESM.zip › DataS4/TmY9qΓèÑ/720575940624591541.pdf]

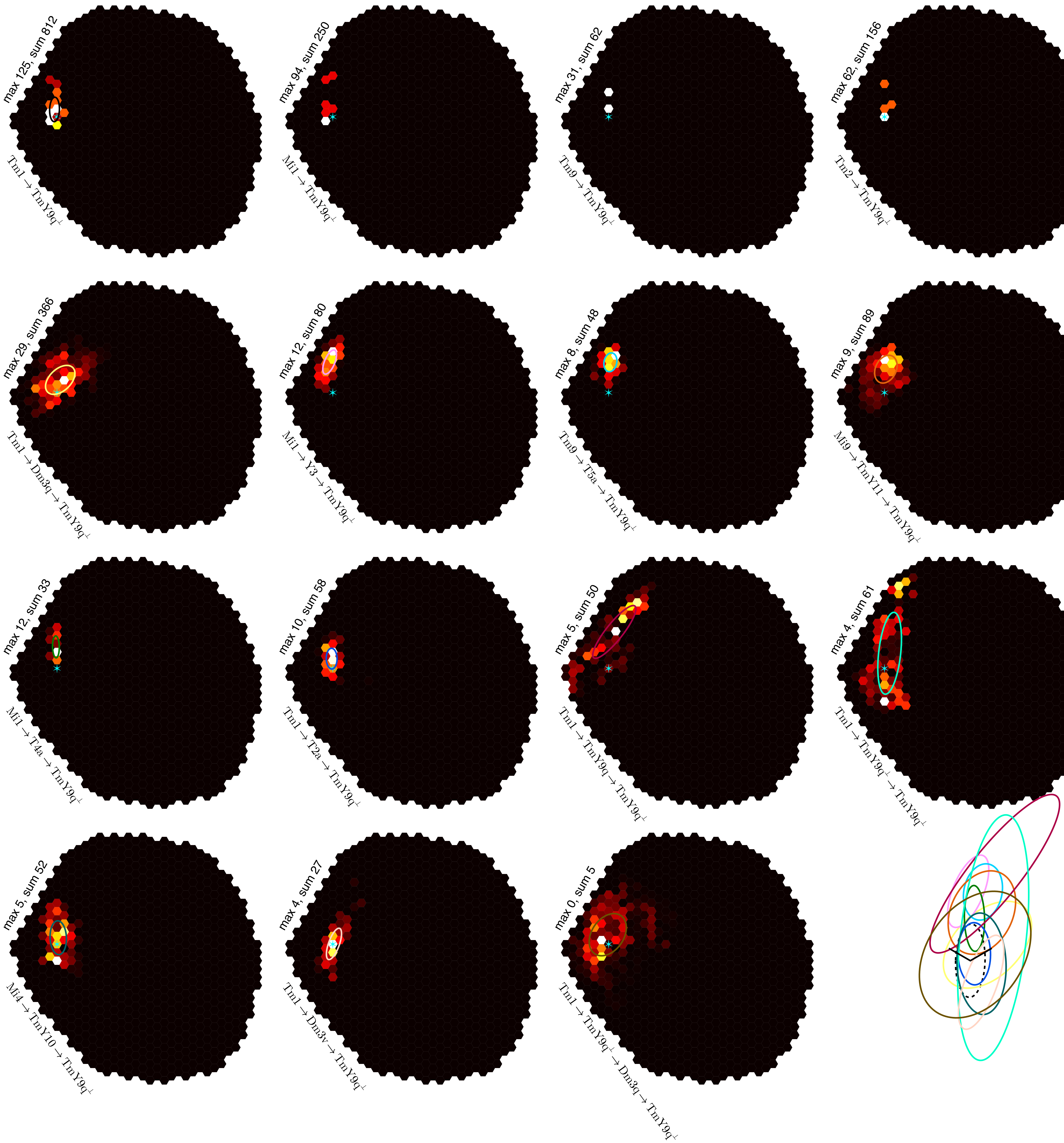
max 125, sum 812
Tm1 → TmY9q⊥
max 94, sum 250
Mi1 → TmY9q⊥
max 31, sum 62
Tm9 → TmY9q⊥
max 62, sum 156
Tm2 → TmY9q⊥
max 29, sum 366
Tm1 → Dm3q → TmY9q⊥
max 12, sum 80
Mi1 → Y3 → TmY9q⊥
max 8, sum 48
Tm9 → T5a → TmY9q⊥
max 9, sum 89
Mi9 → TmY11 → TmY9q⊥
max 12, sum 33
Mi1 → T4a → TmY9q⊥
max 10, sum 58
Tm1 → T2a → TmY9q⊥
max 5, sum 50
Tm1 → TmY9q → TmY9q⊥
max 4, sum 61
Tm1 → TmY9q⊥ → TmY9q⊥
max 5, sum 52
Mi4 → TmY10 → TmY9q⊥
max 4, sum 27
Tm1 → Dm3v → TmY9q⊥
max 0, sum 5
Tm1 → TmY9q⊥ → Dm3q → TmY9q⊥
